# Influence of Corrugated Web Geometry on Mechanical Properties of I-Beam: Laboratory Tests

**DOI:** 10.3390/ma15010277

**Published:** 2021-12-30

**Authors:** Marcin Górecki, Krzysztof Śledziewski

**Affiliations:** Faculty of Civil Engineering and Architecture, Lublin University of Technology, Nadbystrzycka 40, 20-618 Lublin, Poland; k.sledziewski@pollub.pl

**Keywords:** sinusoidal web, geometric parameters of corrugated web, steel I-beam, beam deflection, loss of local stability, loss of global stability

## Abstract

This paper presents the results of experimental investigations performed on beams with corrugated webs. The aim of the research was to determine the effect of the geometric parameters of the sinusoidal web on the behavior of I-beams subjected to four-point bending. Special attention was paid to the effects of web thickness and wave geometry on the deflection of beams. The obtained failure modes of particular test samples are presented. Reference has also been made to the determined standard load capacities based on Annex D of the EC3 standard. In order to compare the performance of beams with corrugated webs, the results for beams with flat webs of the same thickness of web sheets are also presented.

## 1. Introduction

Welded steel girders are mainly I-beams, which consist of a bottom flange, a top flange and a web. All components of the most common plate girders are made from flat plates. It has been shown by the bending analysis of I-beams that the higher the beam height, the greater the bending resistance of the beam. Increasing the beam height also has a positive effect on shear resistance, due to an increase in the cross-sectional area of the web, which is the geometric feature that is responsible. However, the local stability of the web is negatively affected by the action of increasing the web depth. In order to eliminate the problem related to the loss of web stability, the thickness of the web may be increased, or additional stiffeners (transverse and longitudinal) may be applied, in the form of ribs made of flat plates. Note, however, that these measures increase the weight of the structure and significantly increase the cost of manufacture.

**Figure 1:**
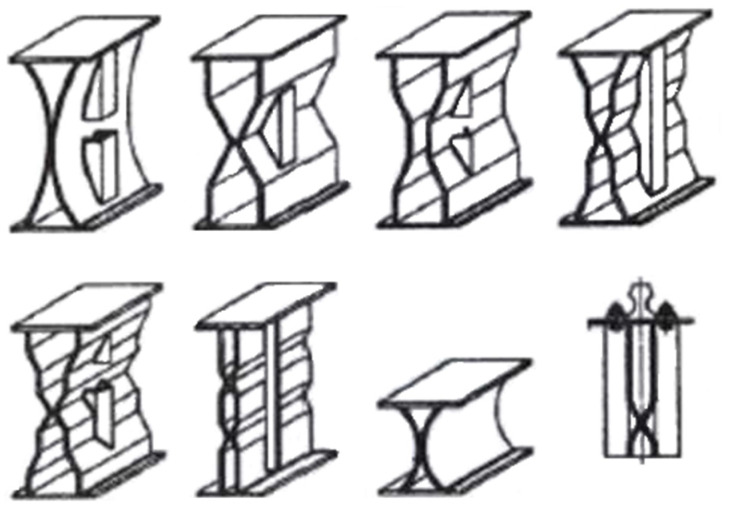
Plate girders with folds parallel to the longitudinal axis of the plate girder [1].

**Figure 2:**
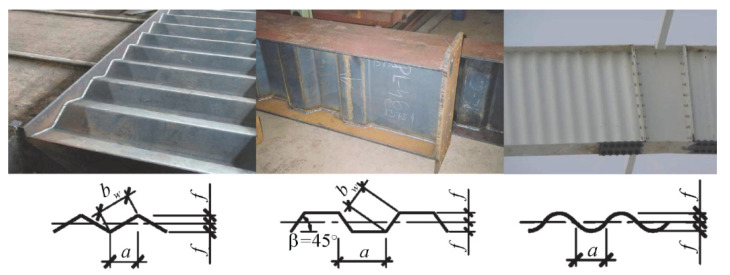
Plate girders with folds perpendicular to the longitudinal axis of the plate girder [2].

Years ago, an important observation was made about the advantages of box profile metal sheeting and the possibility of using it as webs of welded plate girders. Attempts were made to vary the orientation of the corrugation formation in view of the whole girder, even with the crease formation parallel to the longitudinal axis of the plate girder (Figure 1). Plate girders with box profile metal sheeting forming perpendicular to the longitudinal axis of the beam have become very popular (Figure 2).

The process of rippling the web sheeting makes it possible to reduce the thickness of the plate, increases the stiffness of the web and causes an increase in shear stresses. The corrugation of the sheeting provide ribbing in the web. As a result, it leads to the removal of stiffening ribs. This reduces the intrinsic weight of the plate girder by up to 40% compared to a flat web plate girder [3]. To achieve the same shear strength, Papangelis et al. [4] estimated that the amount of steel required to construct a beam with a flat web must be more than double that of a corrugated web.

The production of beams with corrugated webs is mainly based on webs made of trapezoidal sheeting [5,6,7]. Most research is also carried out on this type of plate girders. The sinusoidal webs are also in the sphere of deep interest [8,9,10]. Regardless of the sheet metal used for the web, it has been proved that the contribution of the web to the normal stress transfer is small [11,12,13,14,15]. This was also confirmed by the authors’ own research [16,17,18].

As far as the shear strength is concerned, studies carried out so far [19,20,21,22,23,24,25] have shown that it is provided only by the web. Consequently, the interaction between the bending moment and the shear force can be omitted when determining the shear resistance [26,27,28]. Based on these assumptions, the performance of plate girders with a corrugated web can be compared to that of a truss. Guidelines for determining the resistance of such beams are given in European documents [29,30], which are based on the truss model of plate girder performance.

The paper presents the results of experimental investigations carried out on single-span steel I-beams equipped with webs of corrugated and flat sheets. The systems considered were characterized by the same material properties and geometric dimensions both in terms of cross-sectional area, span and overall length. The ratio of the height to the span of the test samples was 0.1. The beams were tested in a four-point bending scheme.

The main objective of this study was to experimentally determine the effect of the geometry of sinusoidal corrugated webs on the mechanical properties of steel I-beams subjected to four-point bending.

## 2. Experimental Program

### 2.1. Test Specimens

The influence of web geometry on the mechanical properties of the steel plate girder was determined experimentally and based on laboratory tests conducted on eight test pieces. The variables in the performed tests were web type, shape and thickness. The remaining geometric parameters, including material properties, were the same for all samples. The exact dimensions of the tested elements are summarized in Table 1.

**Figure 3:**
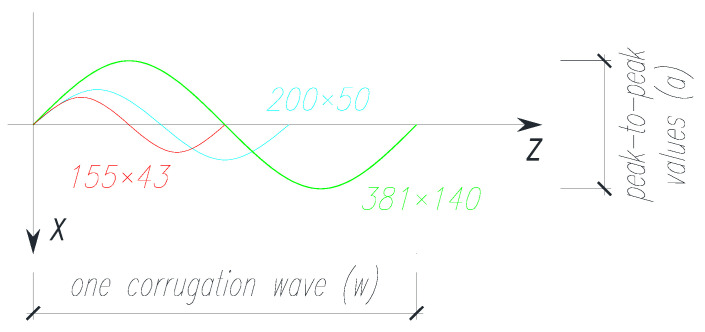
Sinusoidal wave geometry of the considered box profile metal sheeting webs (dimensions in mm).

The main difference of the individual test samples concerned only the webs. Beams with sinusoidal corrugated webs were tested, but also beams with flat webs. The webs were made of sheets of two thicknesses (*t_w_*) 3 mm and 5 mm and of a constant height (*h_w_*), 250 mm. The beams with sinusoidal webs differed in wave amplitude (*a*) and period (*w*), which were 21.5 mm, 27.5 mm and 70 mm, and 155 mm, 200 mm and 381 mm, respectively (Figure 3). For the elements designated as BS 155, two beams with the same web thickness of 3.0 mm were experimentally tested due to the non-availability of the 155×43 sheet profile with a thickness of 5 mm. The first beam of the series was treated as the pilot beam (BS 155/3/1) and the second beam as the main beam (BS 155/3/2).

**Figure 4:**
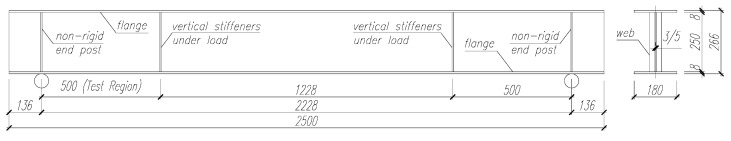
Parameters of experimentally tested beams (dimensions in mm).

Geometric parameters of tested beams are shown in Figure 4. All girders were characterized by a span of 2228 mm, which resulted from the construction of the test stand. In places of support and application of external load, the elements were reinforced with vertical single bilateral stiffeners at full height from flat sheets with a thickness of 5 mm and variable width depending on the shape of the web [31]. Due to negligible values of longitudinal circumferential stresses in the corrugated web panels [32], susceptible support ribs were used [30,33]. The length of the sheet metal end on each side was assumed to be 136 mm, so the total length of the test samples was 2500 mm. The individual components of the plate girders were connected to each other using continuous 4 mm bilateral fillet welds.

### 2.2. Properties of Construction Materials

**Figure 5:**
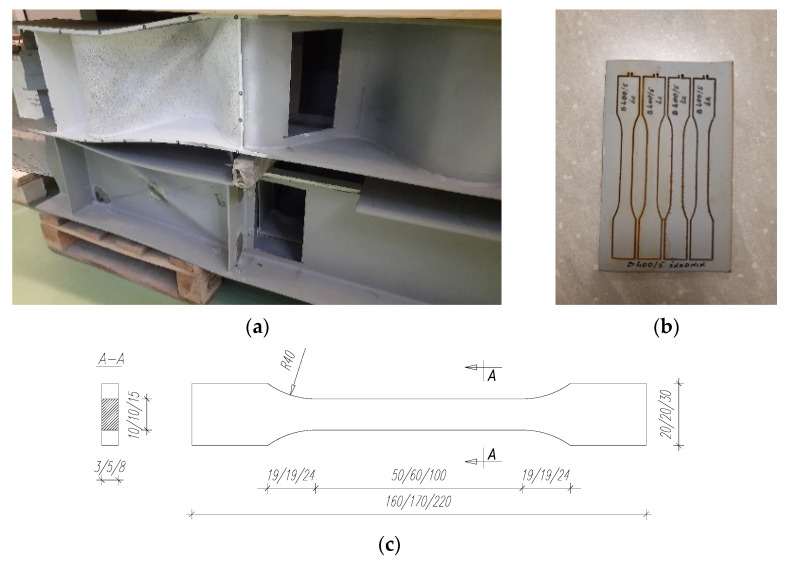
View: (**a**) sampling location for material testing; (**b**) disc from web with cut samples; (**c**) dimensions of the samples depending on the place of sampling: web/web/flange (dimensions in m).

All test samples are made of S355 steel. In order to determine the exact material parameters, additional strength tests were carried out on samples taken from sections of the beams, which had not been subjected to overstrain during the basic test; see Figure 5a,b. The flanges of all the beams were made from a single-delivery sheet, so samples were only taken from one beam. The discs from the beam webs for sample preparation came from the sections where the curvature of the sine wave was closest to a straight section, i.e., from the so-called zero wave inflection.

The dimensions of the samples (Figure 5c) and static tensile tests were carried out according to [34]. Table 2 summarizes the obtained mechanical properties of steel, i.e., tensile strength (*f*_u_) and longitudinal modulus of elasticity (*E*) determined by an extensometer.

### 2.3. Test Implementation

**Figure 6:**
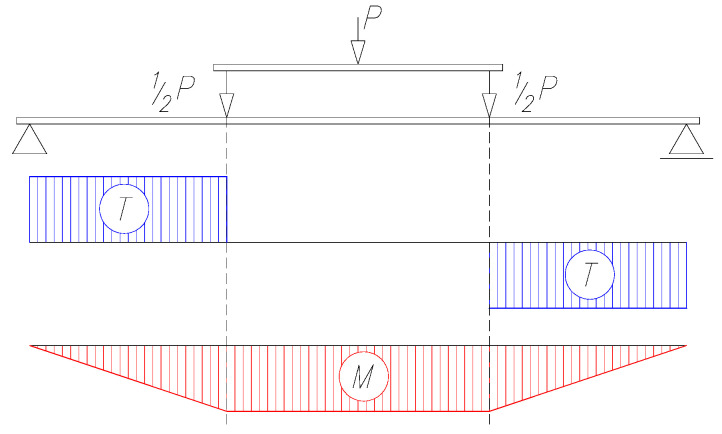
Distribution of bending moments (M) and shear forces in the assumed static scheme.

Destructive tests of beams as well as supplementary material tests were carried out in the accredited Construction Laboratory, which is a unit of the Faculty of Construction and Architecture of the Lublin University of Technology. Beams were tested in the four-point bending scheme (Figure 6).

**Figure 7:**
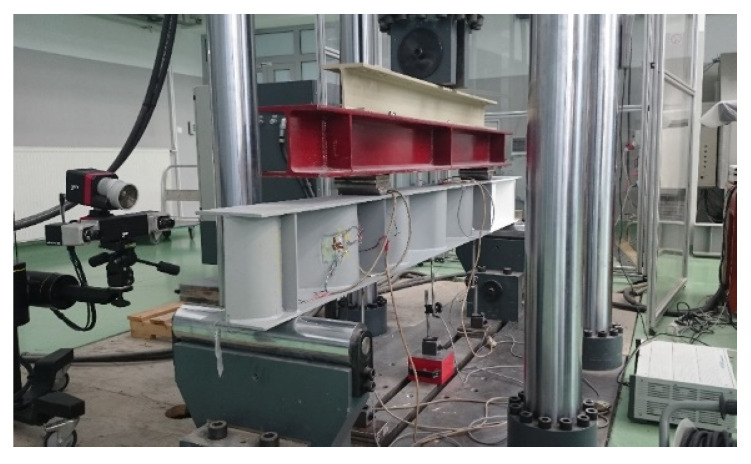
Example of a test sample set up on the test stand.

Figure 7 shows an example of a test sample set up on the test stand. One of the supports was made as a non-sliding pinned support and the other as a sliding pinned support. The loading forces were applied at a distance of 636 mm from each end of the plate. The distance between them and the length of the beam over which the bending moment was constant was 1228 mm.

The external loading was realized by means of one actuator and a special rigid structure consisting of two traverse beams. In addition, the forces were applied to the top flange of the plate girder via a steel half-shaft distributing them to the flange transverse to the beam axis.

The loading process was carried out in “load–unload” cycles. In each successive cycle, the force increased by 50 kN until failure. The exception was the BS 155/3/1 beam, treated as the pilot beam, for which the force increment was slower and increased by 20 kN per cycle. Once the minimum or maximum load was achieved in each cycle, the force was held constant for a period of approximately 180 s. During this time, the readings from the measuring devices were recorded and the changes occurring during the test on the test pieces were observed.

### 2.4. Arrangement of Measuring Sensors

**Figure 8:**
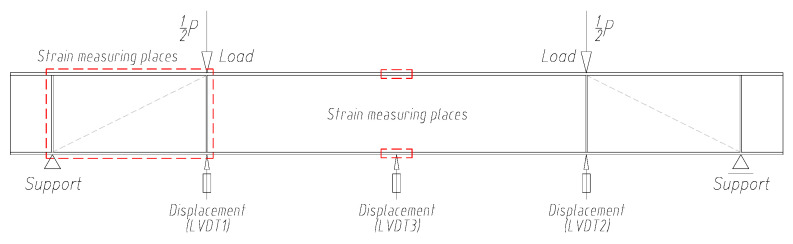
Distribution of displacement sensors and location of strain measurement sites on tested beams.

During the tests, the vertical displacements of the test samples were measured at three points using inductive sensors. The measuring points were located on the underside of the bottom flange directly under the forces and in the middle of the beam span (Figure 8).

**Figure 9:**
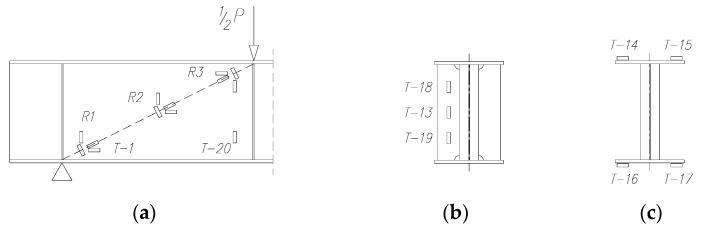
Distribution of strain gauges: (**a**) on the web along the “force–support” line, (**b**) on the vertical rib under the force and (**c**) on the flanges in the middle of the beam span.

The main test area was the web in the shear zone, see Figure 4. The web deformations were measured at three locations using foil strain gauges type TFs-10/120 with 120 Ω resistance and a 10 mm base/length (Figure 9a).

**Figure 10:**
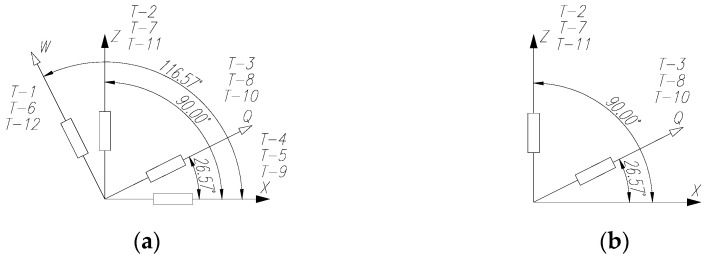
Strain gauge arrangement in the strain gauge ring: (**a**) layout 1 and (**b**) layout 2.

For the pilot beam, four strain gauges were used at each measurement location to form a rosette in arrangement 1 (Figure 10a). On the other beams, two strain gauges were placed on the web near the support and the applied force in arrangement 2 (Figure 10b) and four strain gauges were placed in the middle part of the constant shear force zone (Figure 9a). The angle between each strain gauge was taken from the line formed in the “force–support” direction.

Additionally, deformations were measured on the ribs located under the applied forces (Figure 9b) and on the outer surfaces of the upper and lower flanges, two strain gauges were placed at the mid-span of the beam, just at the outer edges of the flanges (Figure 9c).

## 3. Results and Discussion

### 3.1. Standard Load Capacity of Girders vs. Limit Loads

The standard load capacities of the tested beams were determined according to EN 1993-1-5 Annex D [30] in the case of beams with wavy webs, and to EN 1993-1-1 [34] in the case of beams with flat sheet webs. Because the flanges only transmit internal forces due to bending moments in beams with web corrugations, the bending resistance due to tension of one flange and compression of the other was determined for these test members.

Table 3 shows the load capacity values according to the standard guidelines and the limit load values *P_gr_* of the test samples obtained during the experimental tests. The percentage ratio of the shear force (½*P_gr_*) at failure of the test sample to the shear resistance *V_Rd_* is also shown. The table also shows the percentage ratio of the bending moment *M_ed_* resulting from the limit load *P_gr_* to the bending resistance *M_Rd_*.

Based on the assumptions for the determination of the bending resistance of beams with sinusoidal webs according to EC3 [30], all beams with the same cross-section of flanges and the same web height show the same resistance. For the systems analyzed and assuming the same material parameters, the design moment capacity was (the smallest determined value from *M_Rd_*—in this case, *M_Rd3_*) 124.57 kNm. Actual values of bending moments of the beams were obtained in the range from 82.09 kNm to 144.72 kNm. Only in the case of the BS 200/5 element did the value of the actual moment exceed the standard value, which is confirmed by the form of beam failure obtained during testing.

The resistance to bending, in conventional beams with flat webs, is determined by the strength index of the entire section, among other factors [34]. Therefore, the resistance to the bending (*M_Rd_*) of a beam with a web thickness of 3 mm was 138.39 kNm, and for a beam with a web thickness of 5 mm, it was 145.34 kNm. For these systems, the normal bending resistance was not exceeded.

The shear strength in beams with corrugated webs according to EC3 [30] depends, inter alia, on the loss of local or global stability of the web. The amplitude of the wave and the thickness of the web sheet therefore affect the shear resistance of such an element. For beams with corrugated webs, the shear force obtained in the tests (½*P_gr_*) ranged from 164.17 kN to 289.43 kN. These values were higher than the design resistance (*V_Rd_*), which was also confirmed by the failure modes obtained during the tests. The exception was the BS 381/5 beam, for which the values of the design and actual resistance were 236.61 kN and 249.66 kN, respectively. The failure of this element occurred as a result of flange failure and not web failure, as in the case of other elements.

For the beam with 3 mm-thick flat webs, the shear strength (108.15 kN) was much lower than the shear force induced by the external failure load (150.96 kN). This is also confirmed by the failure form obtained during the experimental tests. Due to the high and similar strain of the element in both shear (94.3%) and bending (97.5%), the beam failure occurred as a result of the interaction of these two forces.

Analyzing the results obtained, it is also found that the beams with webs made of sheets of thickness of 3 mm and similar ratios of wave amplitude to its period (*a/w* in Table 1), i.e., beam BS 155/3 and beam BS 200/3 showed a similar value of the destructive force. However, the value of this force was slightly lower in the case of the BS 200/3 beam. An increase in the *a/w* ratio was not unambiguous, with an increase in the value of the failure load, which is confirmed by the results obtained for the BS 381/3 beam. The lowest value of the failure load was characterized by the BP 250/3 beam with a flat web.

Lack of the BS 155 test sample with a 5 mm-thick web made it impossible to check experimentally whether there was a similar relation between the ratio of wave amplitude to its period and the value of breaking force, as in case of beams with thinner webs. In the case of beams with corrugated webs, an increase in web thickness resulted in an increase of the failure load by a similar percentage—47.3% (BS 200) and 44.1% (BS 381). However, for beams with traditional flat webs, there was an increase in the failure load by as much as 88%, from 150.96 kN (BP 250/3) to 283.42 kN (BP 250/5). This was lower than for the BS 200/5 sample (289.43 kN) but higher than for the BS 381/5 beam (236.61 kN).

### 3.2. Damage Analysis

The research scheme adopted assumed testing of beams in a four-point bending scheme. This allowed the analysis of beam behavior under load-inducing internal forces with a constant bending moment in the central part of the beam and a shear force in the shear zone.

**Figure 11:**
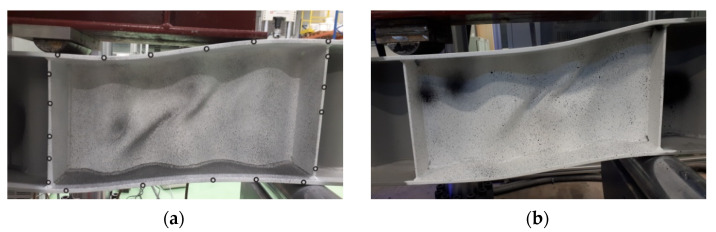
Forms of failure of BS 155 beams with a 3 mm-thick web: (**a**) pilot beam and (**b**) main beam.

**Figure 12:**
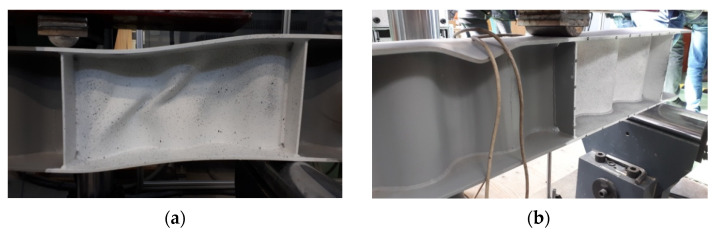
Forms of failure of BS 200 beams: (**a**) 3 mm-thick web and (**b**) 5 mm-thick web.

**Figure 13:**
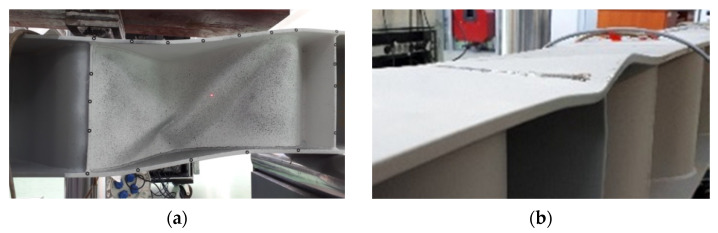
Forms of failure of BS 381 beams: (**a**) 3 mm-thick web and (**b**) 5 mm-thick web.

**Figure 14:**
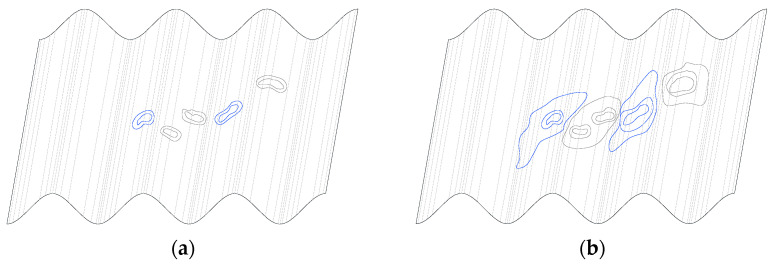
Process of loss of stability of the sinusoidal web: (**a**) formation of foci of local loss of stability and (**b**) formation of changes of direction.

The failure modes of individual test samples with corrugated webs, obtained during the experimental tests, are shown in Figure 11, Figure 12 and Figure 13. In the case of beams equipped with a 3 mm-thick web, the main cause of damage was the loss of web stability. The process of loss of stability of the corrugated web began with the formation of foci of local loss of stability on straight sections between folds (Figure 14a). The local buckling focus led to the formation of plastic changes-of-direction lines approaching the upper flanges (Figure 14b). As a consequence, the flanges collapsed in the beam plane as a result of immediate loading by the transverse force. The experimentally obtained failure modes and load limits for the pilot beam and the main beam in the BS 155 beam group confirm the repeatability of the results.

As observed, for beams with 5 mm-thick corrugated webs, the shear strength utilization ratio took a value less than the resistance-to-bending ratio, see ½*P_gr_/V_Rd_* and *M_Ed_/M_Rd3_* in Table 3. Hence, these beams lost their resistance due to the failure of the top flange, in the zone of constant bending moment value.

**Figure 15:**
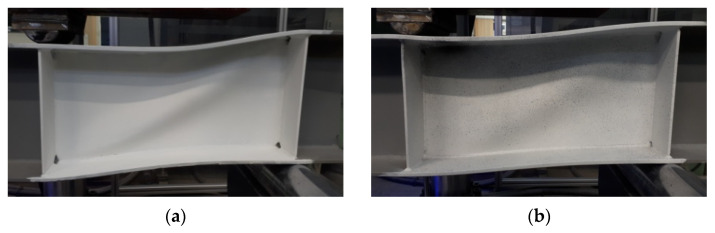
Failure forms of BP 250 beams: (**a**) 3 mm-thick web and (**b**) 5 mm-thick web.

Comparative beams, whose web was made of flat sheet, were damaged as a result of the drawing field in the web, which in turn led to the formation of plastic hinges in the flanges and, consequently, to the failure of the sample (Figure 15).

### 3.3. Force—Displacement Analysis

Table 4 contains the results of vertical displacements of the considered systems. The maximum values of deflections obtained during the tests at the limit load values *P_gr_* for each test sample are presented. Laboratory tests have shown that an increase in the thickness of the web sheet causes an increase in the value of the breaking force with a simultaneous increase in the value of deflections. In order to determine the effect of wave amplitude on the value of deflections of beams, a comparison was made between the change in the deflection of beams with the same wave amplitude but different thicknesses. The results are presented in the form of percentage increments (Δ) of the vertical displacement (at the location of *LVDT 3* sensor) of beams with 5 mm web thickness, with respect to beams with 3 mm web thickness. The table also includes the percentage increment of the limit load Δ*P_gr_* for the same systems.

One of the factors influencing the value of vertical displacements of beams with sinusoidal web is its wave amplitude. The smallest value of vertical deflections was obtained for the test samples having the wavy web with the largest amplitude (140 mm—beams of the BS 381 group), while the largest deflections were observed for the beams with the smallest amplitude (43 mm—BS 155 beams).

However, it should be noted that this increase is small as compared to the increase in the failure load values. In the case of the tested elements, there was a percentage increase in deflection values of 17.4% and 26.3%, with a percentage increase in failure load values of 47.3% and 44.1% for BS 200 and BS 381 beams, respectively. This is a major difference with respect to beams with a flat sheet web, where increasing the web thickness resulted in a percentage increase in a failure load of 87.7%, with a simultaneous percentage increase in deflections of as much as 289.6%.

**Figure 16:**
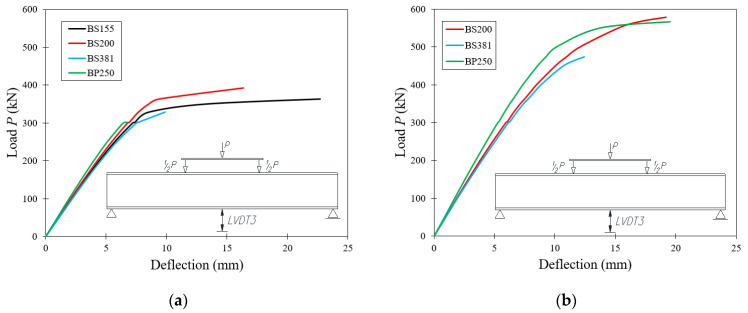
Comparison of deflection obtained during experimental testing of test samples with a web thickness of: (**a**) 3 mm and (**b**) 5 mm.

Figure 16 shows a comparison of the dependence of the deflection at the *LVDT 3* sensor location on the loading force for all the tested elements, with respect to the web thickness.

Beams with flat webs, regardless of web thickness, show less deflection compared to beams with corrugated webs. There is a certain dependence of deflection on limit load: the higher the ultimate force of the test sample, the higher its stiffness. On the basis of the experimentally obtained course of the relation vertical displacement as a function of the applied external force, it can also be observed that an increase in the thickness of the web sheet leads to the obliteration of the point enabling an unambiguous indication of the critical force at which the beam loses stability.

### 3.4. Analysis of the Strain Distribution

The recorded results of strain in particular elements of the tested beams are presented in diagrams depending on the loading force. The strain gauge markings used in the diagrams, as well as the directions in which they were placed, are consistent with those in Figure 9 and Figure 10.

**Figure 17:**
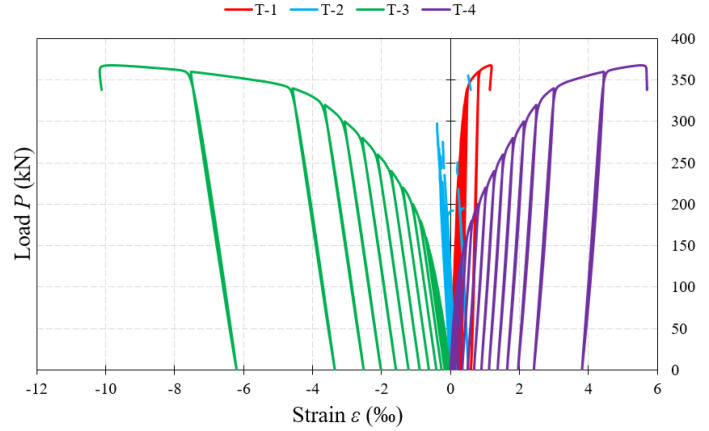
Load–strain relationship in the measurement points (T-2, T-3) located on the web surface at the support.

**Figure 18:**
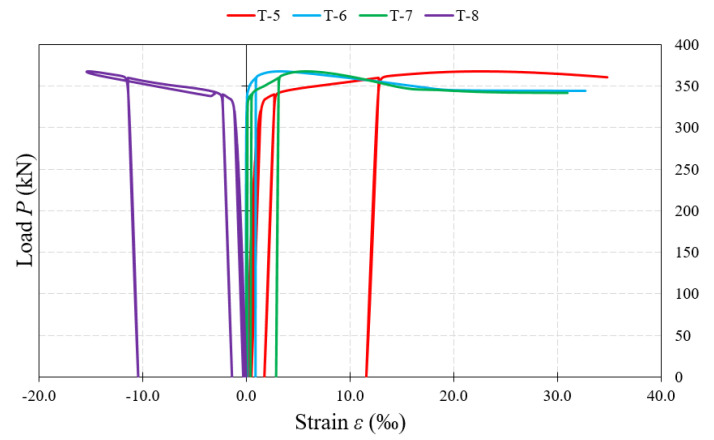
Load–strain relationship in the measurement points (T-5, T-6, T-7, T-8) located on the web surface in the middle part.

**Figure 19:**
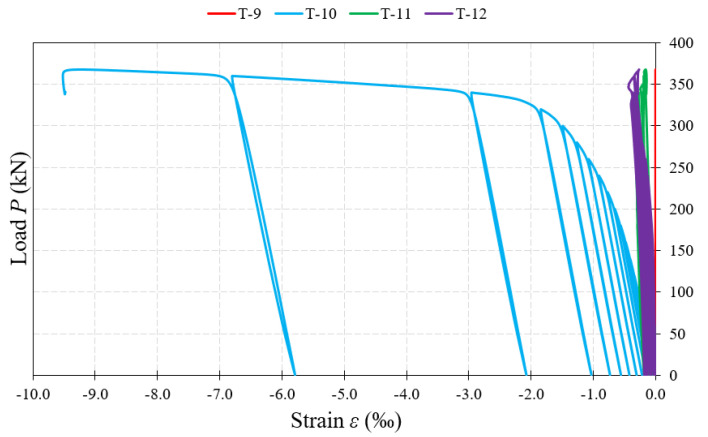
Load–strain relationship at the measurement points (T-10, T-11) located on the web surface at the external force.

Figure 17, Figure 18 and Figure 19 show examples of load–strain relationships obtained at each measurement point located on the surface of the corrugated web along the line defined by the “force–support” straight line. From among all the measuring points, the strain gauges located in the “Q” direction (T-3, T-8, T-10), clearly showed strain values greater than those of strain gauges located on the “W” and “Z” directions. A slightly lower value, in relation to the direction “Q”, was registered by the strain gauges placed on the direction “X”.

The highest strain values, during the process of loading the beams until their complete failure, were shown in most of the test samples by the T-10 strain gauges located under the concentrated force. The exceptions are beams of the BS 155 group, for which, in the initial phase of loading, the value of strain increased faster in the T-3 strain gauges located near the support. In the later loading phase, an accelerated process of strain increase with increasing loading force was observed in the T-8 strain gauges for beams of the BS 155 group and for the BS 200/3 beam. In beams with webs of 3 mm-thick corrugated sheets, after exceeding about 90% of the failure load, a sudden increase in strain was recorded each time, which could indicate the occurrence of a global loss of stability leading to total failure of the test sample.

Further, analyzing the increment of permanent strain with increase of loading force recorded by individual strain gauges, it was observed that it had similar character again for beams of the BS 155 group and the BS 200/3 beam. The highest value of increment was recorded after exceeding about 65–85% of the failure load. In the BS 200/5 and BS 381/3 test samples, a slow increment in strain was recorded at strain gauges close to the support up to a value of about 12% of the failure load. Beyond this point, further strain increment was rectilinear and uniform in each successive cycle.

**Figure 20:**
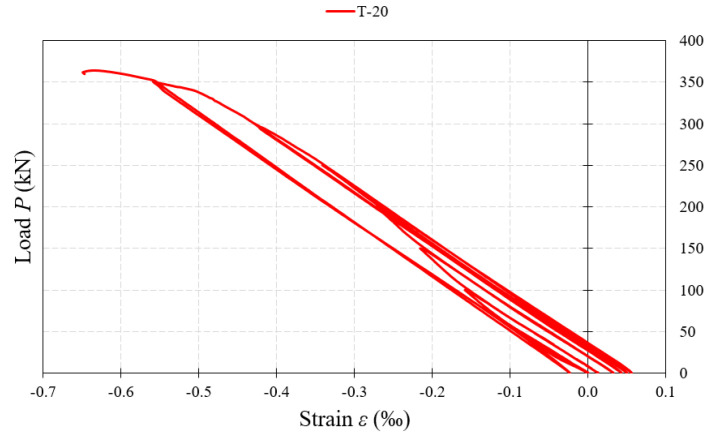
Load–strain relationship at the measurement point (T-20) located on the web surface, at the bottom under the applied force.

T-20 strain gauges placed on the surface of the web, in its lower part under the applied external force, recorded strain indicating the occurrence of compressive stresses (Figure 20). Only in the case of the BS 155/3/2 beam, at the stage of unloading the test sample, a small value of deformation indicating the occurrence of tensile stresses was recorded. The value of deformations at this measurement point in beams with 3 mm-thick corrugated webs was lower than 1‰, whereas in beams with 5 mm-thick webs, it was within the range of 1‰ ÷ 2‰.

**Figure 21:**
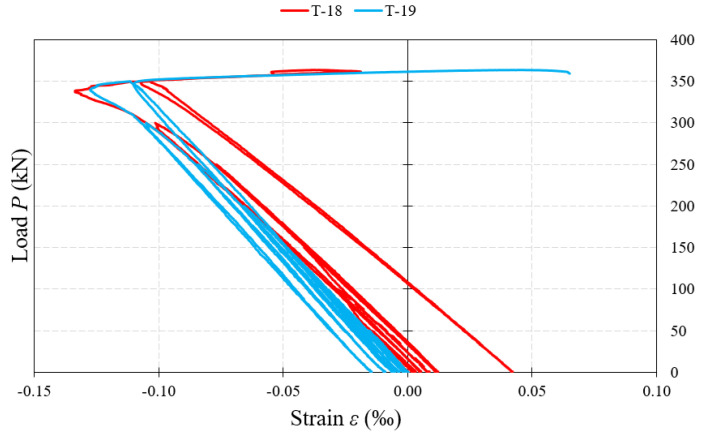
Load–strain relationship at the measurement points (T-18, T-19) located on the rib.

T-18 and T-19 strain gauges measuring deformations on the surface of ribs, near the web under concentrated forces, recorded small values of strain below 0.2‰, see Figure 21. Their course testified mainly to the rib compression and indicated the change of stress type at the maximum values of load in a given cycle and for the total unloading of the beam.

**Figure 22:**
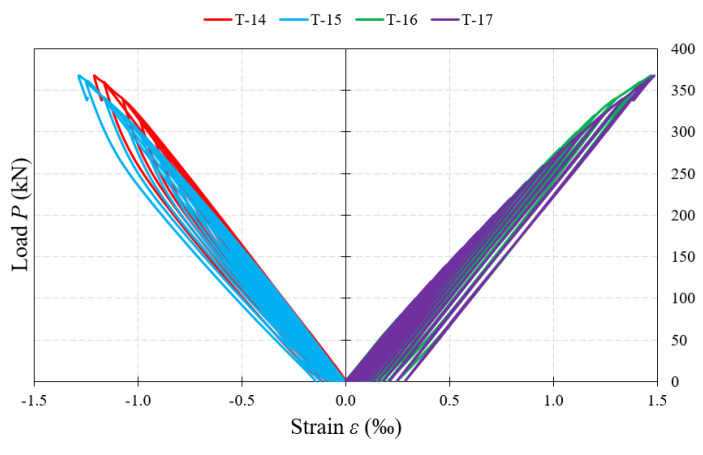
Load–strain relationship in the measurement points located on the upper belt (T-14, T-15) and the lower belt (T-16, T-17).

Strain gauges located on the flanges of the tested beams clearly showed compression on the upper flanges and tension on the lower flanges (Figure 22). In beams with 5 mm-thick webs, at the time of failure, the strain exceeded the value of 2‰.

## 4. Conclusions

Single-span steel I-beams with sinusoidal webs were subjected to tests. The beams were characterized by a different wave amplitude and web thickness. However, the remaining geometric and material parameters were unchanged. The results were compared to identical steel beams with a flat web and to the standard load capacities determined from Annex D of EC3 [30].

The conducted tests and analysis of the obtained results allowed us to formulate the following specific conclusions:

1. The value of the limit load on I-beams is strictly dependent on the web thickness and, in case of beams with sinusoidal web, on the ratio of the wave amplitude (*a*) to its period (*w*). An increase in web thickness results in a significant increase in the value of failure loads for beams, both with sinusoidal and flat webs. On the other hand, an increase in the *a/w* ratio in beams with sinusoidal webs adversely affects the load capacity of such systems. From the group of all test samples subjected to loading with a web thickness of 3 mm, the highest value of failure load was obtained for beams with the most popular web geometry, i.e., 155 × 43, for which the *a/w* ratio = 0.277. It should be noted, however, that the BS 200/3 beam with a similar *a/w* value of *a/w* = 0.275 obtained a similar value of failure load. In beam BS 381/3, for which *a/w* is much higher (*a/w* = 0.367), the failure load was the lowest.
2. The deflection values at the failure load for beams with sinusoidal web depend more on the shape of the sinusoid than the web thickness itself. The deflections of beams subjected to bending indicate that an increase in web thickness by 60% results in an increase in the maximum deflection at failure load. This is evident in the context of the increase in failure load in beams with higher web thickness. The increase in vertical displacement was less than 30% in beams with the same sinusoidal shape but different thicknesses, with an increase in failure load of no more than 50%. Beams with flat webs behave differently. An increase in failure load of less than 90%, for a change in web thickness of 60%, resulted in an increase in vertical displacement at mid-span of about 290%.
  Design of beams with sinusoidal webs, according to EC3 [30], Ref. [30] may not be carried out for all beams of this type regardless of the shape of the sinusoid. The calculations performed showed a lower load capacity than that resulting from the tests for BS 155/3, BS 200/3, BS 200/5 and BS 381/3 beams. The exception was the BS 381/5 beam, for which the shear resistance and bending resistance, according to EC3 [30], were higher than the shear force and bending moment, considered separately; this resulted from the destructive force of the test element. The failure was probably caused by the interaction of the shear force and the bending moment.

## Figures and Tables

**Table 1:** Dimensions of tested plate girders.

Girder	Type of Web ^1^	Web Thickness *t_w_*	Web Height *h_w_*	Peak-to-Peak Values *a*	Wave Period *w*	aw	Flange Dimensions *b_f_ × t_f_*
		(mm)	(mm)	(mm)	(mm)	(–)	(mm)
BS 155/3/1	CW	3	250	43	155	0.277	180 × 8
BS 155/3/2	CW	3	250	43	155	0.277	180 × 8
BS 200/3	CW	3	250	55	200	0.275	180 × 8
BS 200/5	CW	5	250	55	200	0.275	180 × 8
BS 381/3	CW	3	250	140	381	0.367	180 × 8
BS 381/5	CW	5	250	140	381	0.367	180 × 8
BP 250/3	FW	3	250	-	-	-	180 × 8
BP 250/5	FW	5	250	-	-	-	180 × 8

^1^ CW—Corrugated web; FW—flat web.

**Table 2:** Material characteristics of girder steels.

Place of Sampling	Girder	Tensile Strength *f_u_*	Young’s Modulus *E*
		(MPa)	(GPa)
Web	BS 155/3/2	486.80	204.26
Web	BS 200/3	509.21	197.82
Web	BS 200/5	497.42	197.02
Web	BS 381/3	460.39	199.81
Web	BS 381/5	495.97	194.39
Web	BP 250/3	517.45	210.10
Web	BP 250/5	511.74	205.73
Flange	BP 250/3	443.94	180.45

**Table 3:** Determined standard load capacities according to EC3 [30,34] and limit loads obtained during own tests.

Girder	Calculations According to EC3				Laboratory Test			Comparison of Laboratory Test and Calculations According to EC3	
	Shear Resistance of the Web	Bending Resistance			Total Load	Half of the Loading Force	Bending Moment	Degree of Utilization of the Shear Resistance	Degree of Utilization of the Bending Resistance
	*V_Rd_*	*M_Rd1_*	*M_Rd2_*	*M_Rd3_*	*P_gr_*	½*P_gr_*	*M_Ed_*	½*P_gr_/V_Rd_*	*M_Ed_/M_Rd3_*
	(kN)	(kNm)	(kNm)	(kNm)	(kN)	(kN)	(kNm)	(%)	(%)
BS 155/3/1	146.61	131.89	131.89	124.57	367.72	183.86	91.93	125.40	73.80
BS 155/3/2	146.61	131.89	131.89	124.57	363.61	181.80	90.90	124.00	73.00
BS 200/3	142.25	131.89	131.89	124.57	393.01	196.51	98.25	138.10	78.90
BS 200/5	256.20	131.89	131.89	124.57	578.85	289.43	144.72	113.00	116.20
BS 381/3	138.75	131.89	131.89	124.57	328.35	164.17	82.09	118.30	65.90
BS 381/5	249.66	131.89	131.89	124.57	473.22	236.61	118.31	94.80	95.00
BP 250/3	108.15	138.39			301.93	150.96	75.48	139.6	54.50
BP 250/5	300.42	145.34			566.84	283.42	141.71	94.3	97.5

**Table 4:** Deflections of tested beams.

Girder	Measuring Point			Percentage Increase in Deflections Δ*LVDT 3*	Total Load *P_gr_*	Percentage Increase in Loads Δ*P_gr_*
	Under Force *LVDT 1*	Central *LVDT 3*	Under Force *LVDT 2*			
	(mm)	(mm)	(mm)	(%)	(kN)	(%)
BS 155/3/1	22.451	---	22.303	---	367.715	---
BS 155/3/2	20.687	22.686	20.188	---	363.608	---
BS 200/3	14.273	16.371	13.636	17.4	393.012	47.3
BS 200/5	14.344	19.214	13.684		578.851	
BS 381/3	8.434	9.858	7.899	26.3	328.345	44.1
BS 381/5	9.600	12.447	9.618		473.217	
BP 250/3	5.156	6.744	4.943	289.6	301.928	87.7
BP 250/5	16.816	19.531	15.682		566.840	

## Data Availability

Data are available on request at corresponding authors.

## References

[1] Kurkin C.A. (1975). Projektirowanie Swarnych Konstrukcij w Maszinostrojenii.

[2] Solov’ev A.V., Lukin A.O., Alpatov V.Y., Savost’yanov V.N. (2012). Account for Performance of Corrugated Web Beams in the Analysis of Constrained Torsion.

[3] Pasternak H., Kubieniec G. (2010). Plate girders with corrugated webs. J. Civil Eng. Manag..

[4] Papangelis J., Trahair N., Hancock G. (2017). Direct strength method for shear capacity of beams with corrugated webs. J. Constr. Steel Res..

[5] Cafolla J. (1995). Corrugated Webs and Lateral Restraints in Plate Girders for Bridges. Ph.D. Thesis.

[6] Elgaaly M., Seshadri A., Rodriguez R., Ibrahim S. (2000). Bridge girders with corrugated webs. Transp. Res. Rec..

[7] Prathebha P., Jane H.H. (2018). Corrugated web steel girders-A state of the art review. Int. J. Eng. Res. Dev..

[8] Pasternak H., Branka P. (1998). Zum Tragverhalten von Wellstegträgern. Bauingenieur.

[9] Pasternak H., Robra J., Bachmann V. (2009). Wellstegträger mit größeren Stegblechdicken—Fertigungstechnologie und tragverhalten. Bauingenieur.

[10] Nikoomanesh M.R., Goudarzi M.A. (2020). Thin-Walled Structures Experimental and numerical evaluation of shear load capacity for sinusoidal corrugated web girders. Thin-Walled Struct..

[11] Wang S., He J., Liu Y. (2019). Shear behavior of steel I-girder with stiffened corrugated web, Part I: Experimental study. Thin-Walled Struct..

[12] Eldib M.E.A.H. (2009). Shear buckling strength and design of curved corrugated steel webs for bridges. J. Constr. Steel Res..

[13] Huang L., Hikosaka H., Komine K. (2004). Simulation of accordion effect in corrugated steel web with concrete flanges. Comput. Struct..

[14] Ibrahim S.A., El-Dakhakhni W.W., Elgaaly M. (2006). Behavior of bridge girders with corrugated webs under monotonic and cyclic loading. Eng. Struct..

[15] Yi J., Gil H., Youm K., Lee H. (2008). Interactive shear buckling behavior of trapezoidally corrugated steel webs. Eng. Struct..

[16] Górecki M., Pieńko M., Łagoda G. (2018). Numerical analysis of beam with sinusoidally corrugated webs. AIP Conf. Proc..

[17] Górecki M., Śledziewski K. (2020). Experimental Investigation of Impact Concrete Slab on the Bending Behavior of Composite Bridge Girders with Sinusoidal Steel Web. Materials.

[18] Śledziewski K., Górecki M. (2020). Finite Element Analysis of the Stability of a Sinusoidal Web in Steel and Composite. Materials.

[19] Elgaaly M., Seshadri A., Hamilton R.W. (1997). Bending Strength of Steel Beams with Corrugated Webs. J. Struct. Eng..

[20] He J., Wang S., Liu Y., Wang D., Xin H. (2020). Shear behavior of steel I-girder with stiffened corrugated web, Part II: Numerical study. Thin-Walled Struct..

[21] Leblouba M., Junaid M.T., Barakat S., Altoubat S., Maalej M. (2017). Shear buckling and stress distribution in trapezoidal web corrugated steel beams. Thin-Walled Struct..

[22] Driver R.G., Abbas H.H., Sause R. (2006). Shear Behavior of Corrugated Web Bridge Girders. J. Struct. Eng..

[23] Moon J., Yi J., Choi B.H., Lee H.E. (2009). Shear strength and design of trapezoidally corrugated steel webs. J. Constr. Steel Res..

[24] Hassanein M.F., Elkawas A.A., El Hadidy A.M., Elchalakani M. (2017). Shear analysis and design of high-strength steel corrugated web girders for bridge design. Eng. Struct..

[25] Riahi F., Behravesh A., Fard M.Y., Armaghani A. (2018). Shear Buckling Analysis of Steel Flat and Corrugated Web I-girders. KSCE J. Civil Eng..

[26] Sayed-Ahmed E.Y. (2005). Plate girders with corrugated steel webs. Eng. J..

[27] Kövesdi B., Jáger B., Dunai L. (2016). Bending and shear interaction behavior of girders with trapezoidally corrugated webs. J. Constr. Steel Res..

[28] Jáger B., Dunai L., Kövesdi B. (2017). Experimental investigation of the M-V-F interaction behavior of girders with trapezoidally corrugated web. Eng. Struct..

[29] (1990). Träger mit Schlanken Stegen.

[30] (2006). Eurocode 3: Design of Steel Structures—Part. 1-5: Plated Structural Elements.

[31] Kövesdi B., Braun B., Kuhlmann U., Dunai L. (2010). Patch loading resistance of girders with corrugated webs. J. Constr. Steel Res..

[32] Zevallos E., Hassanein M.F., Real E., Mirambell E. (2016). Shear evaluation of tapered bridge girder panels with steel corrugated webs near the supports of continuous bridges. Eng. Struct..

[33] He J., Liu Y., Chen A., Yoda T. (2012). Mechanical behavior and analysis of composite bridges with corrugated steel webs: State-of-the-art. Int. J. Steel Struct..

[34] (2006). Eurocode 3: Design of Steel Structures—Part. 1-1: General Rules and Rules for Buildings.

